# Transcriptomic signatures of subcutaneous adipose tissue in patients with diabetes and coronary artery disease: a pilot study

**DOI:** 10.3389/fcvm.2025.1524605

**Published:** 2025-02-05

**Authors:** Ilias P. Doulamis, Bernard Pan, Aspasia Tzani, Jorge Plutzky, G. William Wong, Ahmet Kilic, Risa M. Wolf

**Affiliations:** ^1^ Division of Cardiac Surgery, Department of Surgery, Johns Hopkins University School of Medicine, Baltimore, MD, United States; ^2^ Department of Pediatrics, Division of Endocrinology, Johns Hopkins University School of Medicine, Baltimore, MD, United States; ^3^ Division of Cardiovascular Medicine, Brigham and Women’s Hospital, Harvard Medical School, Boston, MA, United States; ^4^ Department of Physiology, Center for Metabolism and Obesity Research, Johns Hopkins University School of Medicine, Baltimore, MD, United States

**Keywords:** coronary artery disease, diabetes, subcutaneous fat, transcriptome, RNA seq

## Abstract

The exact role of subcutaneous adipose tissue in the interplay between type 2 diabetes (T2D) and coronary artery disease (CAD) is yet to be determined. A prospective cohort study of adult patients with and without T2D undergoing CABG was performed. Subcutaneous adipose tissue was collected during the procedure and RNA seq analysis was performed. A total of 741 differentially expressed genes (DEGs) were identified (332 up- and 409 down-regulated in the T2D group). Our results demonstrated that pathways related to apoptosis and immune response were significantly dysregulated in the adipose tissue of T2D subjects. The main molecular pathways involved were CXCR, NOTCH, STAT, NFKB1 and FGFR pathways, which have a well-documented role in diabetes and CAD. SPI1 and MTF1 were two novel upstream transcription factors identified which have been suggested to be involved in the inflammatory cascade and insulin regulation in diabetes. Three miRNAs were differentially expressed between the two groups (miR-27a, miR-335 and miR-146). These preliminary results provide fertile ground for further research of potential targets for patients with diabetes and coronary artery disease.

## Introduction

Coronary artery disease (CAD) is the most common cause of mortality in patients with type 2 diabetes (T2D). Despite the well-documented association between obesity and T2D, and the adverse effects of visceral adipose tissue (AT), subcutaneous adipose tissue (ScAT) is believed to have beneficial paracrine and endocrine effects on insulin resistance (1). However, its exact role in the interplay between T2D and CAD is yet to be determined (2). Herein, we sought to investigate changes in the transcriptomic profile of the ScAT associated with T2D in patients with advanced CAD undergoing coronary artery bypass grafting (CABG), compared to individuals without T2D.

## Method

This is a prospective cohort of adult patients with and without T2D undergoing CABG at the Johns Hopkins Hospital (JHH) approved by JHH Institutional Review Board (IRB00318696). Exclusion criteria included immunocompromised patients and patients with type 1 diabetes, active cancer, inflammatory bowel disease or previous cardiac surgery. Patient demographics, comorbidities, medications and preoperative blood work were collected. ScAT samples collected from the sternotomy site at the beginning of the case were immediately frozen and stored in liquid nitrogen. RNA seq was performed as previously described (3). Genes with adjusted False discovery rate (FDR) <0.05 and log2-fold change (>1) were characterized as differentially expressed genes (DEGs) for each comparison. MetaCore (v20.2), Appyter (v0.2.6) and GSEA were used for functional enrichment and pathway analysis. STRING (12.0) was used for protein-protein interaction networks. Mutliomic analysis to visualize the miRNA-gene networks was performed in miRNet (2.0).

## Results

**Figure 1:**
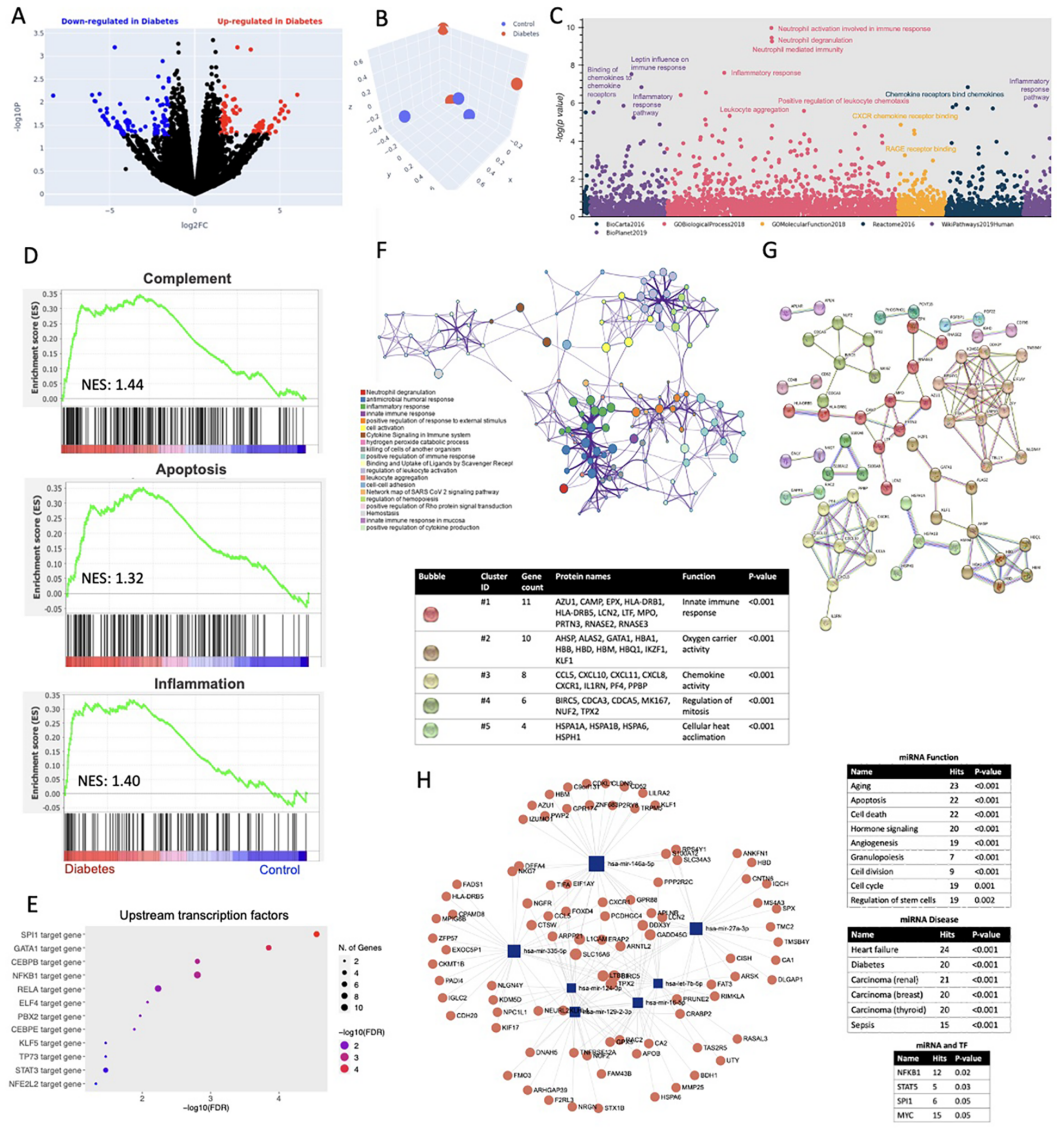
**(A)** Volcano plot indicating the upregulated (red) and downregulated (blue) genes in the diabetes group compared to control. **(B)** Principal component analysis (PCA) for control (blue) and diabetes (red) groups. **(C)** Manhattan plot of six databases indicating the most significant Gene Ontology (GO) molecular functions when comparing diabetes to control groups. **(D)** Enrichment plot analysis indicating upregulated pathways in the diabetes group compared to control. NES: Normalized enrichment score. **(E)** Forest plot indicating significantly upregulated transcription factors in the diabetes group compared to control. FDR: False discovery rate. **(F)** Network analysis of enriched pathways associated with diabetes in our study population. **(G)** Protein string interaction network analysis showing the most significant clusters when comparing diabetes group to control. **(H)** miRNA and transcription factor (TF) interaction analysis network.

A total of 6 patients were enrolled, 3 with T2D (mean age 60.3 years, mean BMI 29.26 kg/m^2^, all with 4-vessel disease, and 2 with HTN, 1 with hyperlipidemia) and 3 without T2D (mean age 65 years, mean BMI 37.07 kg/m^2^, all with HTN and hyperlipidemia, and 4-vessel disease). To ascertain the global transcriptomic changes associated with T2D in the ScAT of these patients, we performed RNA sequencing and gene ontology (GO) pathway analysis. A total of 741 differentially expressed genes (DEGs) were identified (332 up- and 409 down-regulated in the T2D group) (Figure 1A). Principal component analysis (PCA) indicated that the transcriptomic profiles of the two groups were separate and discrete (Figure 1B). Cumulative GO analysis of 6 different databanks showed that the top upregulated pathways in T2D included leptin influence on immune response, inflammatory response to chemokines, leukocyte aggregation, neutrophil activation, CXC receptor binding and heat sock factor 1 (HSF-1) activation (Figure 1C). GSEA enrichment analysis identified apoptosis, inflammation and complement activation to be significantly upregulated in the T2D group (Figure 1D). Transcription factors (TF) PU.1 (SPI1), erythroid transcription factor (GATA), nuclear factor kappa B subunit 1 (NFKB1), signal transducer and activator of transcription factor 3 (STAT3), E74 like ETS transcription factor 4 (ELF4) were found to be upstream regulators of the activated pathways among others (Figure 1E). The network of the most significantly enriched pathways in T2D and their subsequent subclusters are better depicted in Figure 1F indicating the presence of neutrophil degranulation, inflammatory and immune response. Further GO analysis of the top downregulated targets showed that NOTCH signaling and fibroblast growth factor receptor (FGFR) signaling pathways were significantly downregulated in the diabetic patients, while the top predicted upstream regulators for those pathways were found to be the microphthalmia transcription factor 1 (MTF1) -a regulator of beta pancreatic cells- and the fibroblast growth factor receptor 3b (FGFR3b) -a receptor found to be dysregulated in visceral obesity (data not shown). Protein-protein interaction network analysis of the enriched targets indicated that the top 5 clusters are associated with innate immune response, oxygen carrier activity, chemokine activity, regulation of mitosis and cellular heat acclimation (Figure 1G). Multiomic analysis of the top dysregulated miRNAs and proteins in the diabetes group showed that 3 mRNAs, miR-335, miR-27a and miR-146a formed networks with targets associated with pathways of aging, apoptosis and cell death; while the associated disease processes included heart failure, diabetes and different types of cancer. The transcription factors regulating those miRNAs include NFKB1, STAT5 and MYC (Figure 1H).

## Discussion

This is a pilot study investigating the transcriptomic shifts of ScAT associated with T2D in patients with advanced CAD requiring surgical revascularization. It is reported that ScAT potentially exerts a protective role against glucose homeostasis dysregulation. Our results showed that pathways related to apoptosis and immune response were significantly dysregulated in the adipose tissue of T2D subjects. The association between these pathways and the pathogenesis of CAD and T2D through modulation of insulin resistance and low-grade inflammation among others has been previously studied. The main molecular pathways involved in our study, namely CXCR, NOTCH, STAT, NFKB1 and FGFR pathways, have a well-documented role in diabetes and CAD (3). SPI1 and MTF1 were two novel upstream *Τ*Fs identified which have been suggested to be involved in the inflammatory cascade and insulin regulation in diabetes (4). Moreover, three miRNAs were differentially expressed in the adipose tissue of diabetic patients. Interestingly, miR-27a has been previously described as a negative regulator of adipogenesis and is increased by white adipose tissue inflamation (5). However, the role of miR-335 and miR-146 has not been explored beyond inflammation and oncogenesis suggesting a possible link of those miRNAs to targets regulating the inflammatory response and secretion of diabetic adipocytes. Our findings suggest that ScAT is an active paracrine organ which is involved in the low-grade inflammation associated with CAD and is modulated by T2D. Thus, development of drugs that can target these miRNAs could potentially decrease the proinflammatory environment promoted by T2D and CAD and lead to decreased risk for comorbidities and complications.

The extrapolation of our results may be limited by the relatively small sample size which did not allow for subgroup analyses based on further comorbidities or medications. The nature of the current study is exploratory and was not focused on solid comparisons between the two patient populations. Additionally, these are patients with advanced CAD and their transcriptome may differ from those with earlier stages of the disease.

Our next steps are focused on different sources of adipose tissue including perivascular and epicardial fat since their proximity to the heart may unveil new pathways involved in CAD and insulin hemostasis.

## Data Availability

The RNA sequencing data analysed in this study was obtained from Novogene Corporation Inc, the following restrictions apply: data are deleted 30 calendar days after release to the customer. Requests to access the other datasets, including DEGs should be directed to ID, doulamis.i@gmail.com.
